# An internet of radiation sensor system (IoRSS) to detect radioactive sources out of regulatory control

**DOI:** 10.1038/s41598-022-11264-y

**Published:** 2022-05-03

**Authors:** Vinh Tran-Quang, Hung Dao-Viet

**Affiliations:** Hanoi University of Science and Technology, Hanoi, Vietnam

**Keywords:** Engineering, Electrical and electronic engineering, Experimental nuclear physics

## Abstract

A radioactive source that is not under regulatory control, either because it has never been under regulatory control or because it has been abandoned, lost, misplaced, stolen, or otherwise transferred without proper authorization, is considered an orphan source. Orphan sources are usually gathered as scrap metal because of their heavy metallic containers. Melting an orphan source with scrap metal produces contaminated recycled metal and waste; the consequences will be extremely serious for humans and the environment, affecting the economy and social stability. In this paper, we propose and develop an Internet of Radiation Sensor System (IoRSS) to detect radioactive sources out of regulatory control in scrap metal recycling and production facilities. It is a complete IoT system consisting of a network of wirelessly connected radiometric devices that optimizes the detection, localization, and identification of radioactive sources by integrating data from multiple portable radiation detectors. The proposed IoRSS system creates a robust and flexible network architecture along with advanced data fusion algorithms that combine information from many detectors. The IoRSS system provides advanced search and monitoring capabilities in a large coverage area and in difficult operational environments.

## Introduction

Radiation source security, in particular, and nuclear power security, in general, are currently the main concerns of the international community^1^. In recent years, radiation and nuclear technologies have been deployed rapidly and broadly in various industrial and economic sectors and society, which has brought various practical benefits. However, the management, transportation, storage, and usage of radiation sources are complicated by many challenges. In fact, there have been many radiation sources lost, resulting in significant impacts on economic and social stability^2^. A radioactive source that is not under regulatory control, either because it has never been under regulatory control or because it has been abandoned, lost, misplaced, stolen or otherwise transferred without proper authorization, is called an orphan source^3^. Orphan sources have led to accidents with serious, even fatal, consequences as a result of the exposure of individuals to radiation^4^.

The melting of an orphan source with scrap metal or its rupturing, when mixed with scrap metal, has also resulted in contaminated recycled metal and wastes^4^. If this happens, costly cleanup operations may be necessary. If the contaminated material is not detected at the metal recycling and production facility, workers may be exposed to radiation, and radionuclides may become incorporated into various finished products and wastes, which, in turn, may lead to exposure to users of these products. Concern over accidents involving orphan sources, including those that have occurred in the metal recycling and production industries, led to the establishment of an international undertaking Code of Conduct on the Safety and Security of Radioactive Sources (Code of Conduct)^5^. In the general principles section of the Code, it is also stated that each country must have technical systems in place to respond quickly with the goal of controlling stolen and abandoned radioactive sources and eliminating or minimizing their consequences. Nevertheless, the possibility of orphan sources being present in scrap metal remains^6^. Lost radioactive sources are usually sealed sources, made of metal rods and pellets, and their containers are also metal. Therefore, when the radioactive source is lost, it will usually be sold to a steel scrap collector for recycling^2,6–8^. This is the reason why all countries are very interested in controlling radioactive sources in scrap metal recycling facilities. The IAEA has technical guidelines for dealing with this in its document^1^ “Control of Orphan Sources and Other Radioactive Material in the Metal Recycling and Production Industries” (Specific Safety Guide, No. SSG-17, Vienna, 2012). Radioactive and nuclear materials can constitute a threat to public health and homeland security in the form of threats of terrorism, orphan sources, nuclear accidents, or radioactive contamination^9^. As radiation detectors installed at major ports of entry are a key component of the overall strategy to protect countries from nuclear terrorism^10^. In Vietnam, there are also regulations with the responsibility for detecting radioactive sources out of regulatory control for scrap metal recycling and production facilities^7^.

With the advancement of science and technology, especially in the field of nuclear detection technologies, many specialized technologies and equipment have been developed to ensure the safety and security of radioactive sources such as radiation portal monitors (RPMs), personal radiation detectors (PRDs), hand-held radioisotope identification devices (RIIDs), mobile and transportable detectors, radiographic imaging systems employ x-rays or gamma rays^11^. These devices operate individually, have high operating and maintenance costs, and are not suitable for small and medium-sized scrap metal recycling facilities. Another challenge is that when orphan sources are hidden in scrap metal that shields their activity from traditional detectors in the portals that scan incoming trucks^12^.

The detection of radioactive material in waste is of paramount importance for the protection of the environment^12^. In this report, we present the proposal and development of an Internet of Radiation Sensor System (IoRSS) to enhance the use of nuclear detection systems to detect nuclear and other radioactive materials out of regulatory control at points of entry/exit and other trade locations of scrap metal recycling and production facilities. To maximize the ability to detect, identify, locate, and respond to nuclear radiation incidents, we propose and apply advances in computing, communications, algorithm development, software tools, and hardware in an integrated network of distributed sensors^13–16^ and LoRa^17,18^ wireless communications that contribute to enhanced radiological and nuclear detection capability and response activities. The implementation of the IoRSS has facilitated improved situational awareness and better capabilities to detect, identify, locate, and respond to incidents by integrating data from multiple fixed and mobile radiation detection devices across distributed detectors and apply advanced data processing algorithms.

The main contributions and novelty of the paper are given below.

- Propose an Internet of Radiation Sensor System (IoRSS) to detect radioactive sources out of regulatory control in scrap iron and steel recycling facilities. The IoT-based IoRSS system includes a network of wirelessly connected stationary and mobile devices, data processing algorithm and software, monitoring and control servers, web-based and mobile applications, and procedures of radiation incident detection and response plan. The IoRSS system provides a more robust detection capability, more quickly and accurately with high confidence in the presence, location, and type of radioactive material than a group of individual detectors.
- Design and comprehensive field tests of a wireless, compact and robust gamma-sensitive detector for gamma and neutron detection compatible with stationary and mobile devices that are compatible and affordable for installation in several stations or quickly moving to different areas of the scrap yard to scan scrap metal in recycling and production facilities (operation at high temperature and intense vibrations and mechanical shocks) prior to fusion. The design of the proposed IoRSS is fully modularized; therefore, it can be easily customized not only for detectors and other hardware but also for the IoT network and protocol. This makes the system more flexible and feasible.
- Provide radiation detection, identification, warning, and incident response plan. These processes are designed, developed, and integrated into the IoRSS system. In these processes, the stationary device can continuously measure gamma-ray and sending the measurement to the cloud/server. The application server performs data analysis algorithms on the received data. In case the measured value exceeds a predefined threshold, the application server will generate a primary warning so that users can use the mobile device to confirm the existence of the radiation source, identify the type of radiation, the type of radioisotope and the exact location of the radiation source. Based on the confirmed radiation dose rate compared to the level of preconfigured thresholds, the IoRSS system will activate an corresponding incident response to the level of danger of the detected radiation.

The remainder of this paper is organized as follows. “literature review” section highlights the state-of-the-art literature review on aspects of the IoT approach for radiation detection and monitoring, searching and locating lost radioactive sources, and radioactive and nuclear material detectors. The detailed proposal for an IoT-based radiation sensor system (IoRSS), including the system architecture and hardware design, is described in “system architecture and hardware design for an IoT-based radiation sensor system (IoRSS)” section. The processes of radiation detection, identification, warning, and response to radiation incident are described in “IoRSS operation protocols” section. In “test results and analysis” section, we present the results of extensive field tests in scrap metal recycling and production facilities to evaluate the performance of the proposal. The experimental setup and results findings are also extensively analyzed and discussed in this section. Finally, our conclusions and future work are described in “conclusions and future work” section.

## Literature review

### IoT approach for radiation detection and monitoring

The Internet of Things (IoT) is one of the most emerging technologies today and has begun to participate in almost every aspect of our social life, ranging from financial transactions to the healthcare system, communication to national security, battlefields to smart homes, and so on^19^. With a wide range of application domains and high-density IoT network predictions^20,21^, new and complex requirements arise, which need a reassessment of resource allocation and connectivity to enable devices to be deployed to transmit data of IoT applications^22,23^. To increase the performance and reliability of measurements in IoT solutions, the authors in^24–26^ proposed the concept of collaborative IoT-gateways to manage Internet-working connections between devices, other subsystems, and the connection to the cloud. However, the wide deployment of IoT also suffers certain issues, such as interoperability, compatibility^27^, and processing of a large amount of heterogeneous data^24^. The conventional data storage and security mechanisms that are currently in use appear to not be suitable for such a huge amount of generated data in the IoT system. Therefore, the authors in^28^ propose a public-permissioned blockchain security mechanism using an elliptic curve crypto (ECC) digital signature that supports a distributed ledger database (server) to provide an immutable security solution, transaction transparency and prevent patient records tampering at the IoT fog layer. In^19^, the researcher exploits the potential benefits of a blockchain system and integrates it with software-defined networking (SDN) while justifying energy and security issues. The blockchain technology approach also helps mitigate these issues of latency, centralization, and scalability in the fog model^28^.

The consequences of nuclear accidents are enormous, and there have been many nuclear incidents throughout the world that have caused serious consequences for many years afterward. Examples include uncontrolled contamination of the land surrounding the Chernobyl nuclear power plant incident or the damage caused by the tsunami at the Fukushima Daiichi nuclear power plant in 2011, in Japan^9^. With the IoT approach for radiation detection and monitoring, the authors in^29^ presents a real-time surveillance and management system for the security of radioactive material (BKRAD) for security of radioactive source in Vietnam. The BKRAD integrates various positioning and sensing techniques that allow us to continuously monitor radiation source devices under a variety of challenging environmental conditions. The mobile source tracking project in USA^30^ is part of the “Initiative for reducing the global threat”. The mission of this initiative is to minimize risks during the storage, transportation, and operation of mobile radiation sources and to protect vulnerable radioactive and nuclear materials placed in civilian locations around the world. The IAEA develops the radiation safety information management system (RASIMS)^31^. This is a web-based platform that enables member states and the IAEA secretariat to jointly collect, analyze, and view information regarding the national infrastructure for radiation and waste safety. In addition to facilitating the identification of national and regional needs, the information contained in RASIMS is used for a range of other purposes, including the design of new technical cooperation projects and in the radiation safety clearance process prior to the provision of radiation sources to member states.

### Searching and locating for lost and orphan radioactive sources

Locating lost radioactive sources quickly, precisely, and safely is essential in emergency responses of lost radioactive source accidents^9,32–35^. Brunelli et al. proposed DRAGoN^9^, a Drone for RAdiation detection of Gammas and Neutrons, with the goal of designing, developing, and characterizing a mobile system composed of an unmanned aerial vehicle (UAV). The UAV is equipped with a detection system capable of identifying radioactive contamination spread over a few to tens of square meters, which are used mainly in accident scenarios where doses are too high for human safety or in areas of difficult access^9,36,37^. The authors in^32^ describe a source localization approach using an independently developed UAV radiation monitoring system, which uses a specialized source localization algorithm developed on the basis of the inverse-square law and statistical methods. Pavlovsky et al.^34^ proposed a localization and mapping platform (LAMP) to fuse three-dimensional (3D), real-time volumetric reconstructions of radiation sources with contextual information (e.g. LIDAR, camera, etc.) derived from the environment around the detector system. This information, particularly when obtained in real time, can be transformative for applications, including directed search for lost or stolen sources, management after the release of radioactive materials, or avoidance of contamination in security-related or emergency response scenarios^34^. Radioactive sources such as neutrons and gamma emitters can be detected in the proposed drone, which is designed to perform autonomous missions^9^. In^12^, a radiation detection backpack that can be used discreetly or by a wide range of users, was developed using a silicon photomultiplier array (SiPM) and CsI(Tl)^12^, and its characteristics were evaluated. In^38,39^ the authors present a distributed data acquisition (DAQ) system for experimental nuclear physics, in which each task related to the different parts of the DAQ (acquisition, preprocess, analysis, etc.) is run in a separate process to distribute the computational load, giving excellent results in terms of performance and stability. In^10^, distributed mobile sensors, using vehicle platforms, have been proposed to detect nuclear materials in transit in New York City using a combination of radiation transport, and geographic information systems. The results show that the time to first detection increases with the source velocity, decreases with the number of mobile detectors, and reaches a plateau that depends on the strength of the source.

### Radioactive and nuclear materials detectors

Radioactive sources are largely used in industrial and medical applications, and they can be accidentally or intentionally disposed in waste. In particular, a very critical case concerns the presence of radioactive sources in scrap metal^1,4,6,9^. The most common isotopes, with long decay times, that are searched for in scrap metal are ^137^Cs (662 keV) and ^60^Co (1170 and 1330 keV), since they are extensively used in the form of sealed gamma-ray sources in industrial applications, such as sterilization of food and, along with ^192^Ir and ^75^Se, tomography, in particular of high density materials, as in metallurgy and welding verification^8^.

Frequently, radiation monitoring systems, equipped with semiconductor detectors, proportional counters, Geiger-Müller (GM) tubes^40^ or scintillator detectors such as NaI(Tl)^32,33^; and CsI(Tl)^12^, are specifically designed for gamma ray or neutron detection. The use of a single neutron/gamma detector is an interesting solution for detecting and identifying gamma emitters and special nuclear materials (SNM). In^41^, the authors present a comprehensive characterization of a medium size (2″ × 2″) CLLB scintillation detector, to provide the necessary information to assess its deployment in applications regarding homeland security and radiation monitoring. However, it is important to note from^42^ that neutron and gamma measurements are complementary, in particular for the special nuclear materials (SNM) detection, especially when masked or shielded, gamma rays and neutrons have to be detected at the same time in order to increase the sensitivity against natural background. Therefore, finding a unique unit to detect both types of particles is an excellent solution^42^. In literature, several authors have reported the performance of small-sized CLLB scintillators^34,42–44^. However, taking into account that for radiation monitoring (and for other applications) a higher gamma and neutron detection efficiencies would be desired. Woolf et al.^43^ report the results of an experiment conducted to identify isotopic contaminants that produce measurable background emission in a family of inorganic scintillation crystals known as elpasolites, namely Cs_2_LiYCl_6_:Ce (CLYC), Cs_2_LiLaBr_6_:Ce (CLLB), and Cs_2_LiLa(Br, Cl)_6_:Ce (CLLBC), and in other inorganic scintillation crystals, such as Li co-doped NaI:Tl (NaIL). The CLYC scintillator has been shown to be suitable for dual γ-ray/neutron detection owing to its distinct response to both radiation types^44^. In^34^, Pavlovsky et al. present the expansion of these gamma-ray mapping concepts to neutron source localization. This is achieved by integrating a localization and mapping platform (LAMP) with a custom CLLBC scintillator detector sensitive to both gamma rays and neutrons. The European project entitled “effective container inspection at BORDer control points” (C-BORD) focuses on the development and in situ tests of a comprehensive cost-effective solution for generalized Non-Intrusive Inspection (NII) of containers and large volume freight at the European Union (EU) border^39^. The project copes with a wide range of targets, including explosives, chemical warfare agents, illicit drugs, tobacco, and special nuclear materials (SNM). The detection of SNM such as plutonium samples has been tested with the mobile SMANDRA inspection system^42^ both as a high-sensitivity passive spectroscopic system and as a complete active inspection system using tagged neutrons. The test results in^42^ show that active interrogation with tagged neutrons can provide signatures for the discrimination of uranium against other heavy metals.

## System architecture and hardware design for an IoT-based radiation sensor system (IoRSS)

### System architecture and components

**Figure 1:**
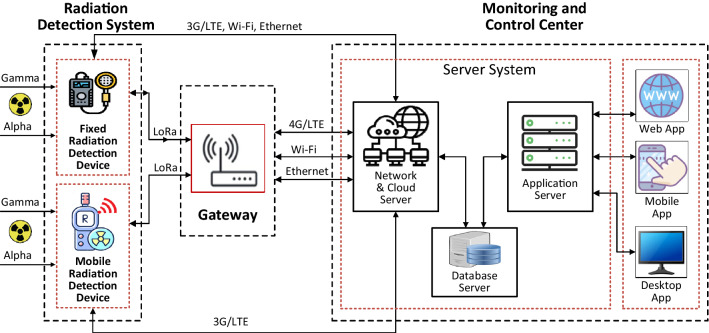
Architecture and components of the IoT based radiation sensor system (IoRSS).

The overall architecture of the IoRSS as depicted in Fig. 1 consists of the following components:

- Fixed radiation detection device (fixed device): This is the stationary device integrated with large, passive detectors, and a wide energy range of measurements for gamma detection and neutron detection. Stationary devices are mounted at points of entry/exit and other trade locations of scrap metal recycling and production facilities to scan large scrap vehicles such as cars and trucks. Operationally, the stationary devices scan for the presence of radiation and are typically coupled with mobile radiation detection devices used in a secondary scanning mode to identify radiation sources. The stationary devices are equipped with wireless (3G/LTE, LoRa) and wired (Internet/WiFi) communications, sensor systems, and other actuators to support their operation and information sharing among system components.
- Mobile radiation detection device (mobile device): This is a portable and transportable detector that generally uses gamma-sensitive detectors for gamma and neutron detection. They can be used as a handheld device for area surveillance, search, or other temporary deployments, such as between metal scrap yards and vehicle entrance/exit gates, or in smelting preparation areas. This device also has built-in radioisotope capabilities suitable for isotopic identification of nuclear and radioactive materials. Operationally, the handheld device is typically coupled with a fixed radiation detection device used in a secondary scanning mode to identify radiation sources and to confirm for activating corresponding incident response procedures. The handheld device is integrated wireless communication technologies, such as LoRa and ZigBee to communicate with a Gateway and 3G/LTE to communicate with the network and cloud server. The mobile radiation detection device is designed with compact size, is portable, and is easy to move, is capable of mounting on an unmanned aerial vehicle (UAV), and is suitable for the tasks of searching for radioactive sources in spread out, unfocused space or radioactively contaminated areas.
- The centralized gateway device (gateway): This device supports multiple wireless connection platforms to communicate with fixed/mobile devices, allowing data to be received from the devices, performing processing functions, combining data, and transferring data to the network and cloud server.
- The monitoring and control center (servers): This is a system of servers and software tools that support information exchange, protocol development, design advanced data fusion algorithms that combine information from many detectors, automate information processing to optimize limited resources, and improve data analysis in radiation incident assessment and response plans. The servers also provide services to develop applications for end-users.

The radiation detection subsystem consists of two types of independent devices: fixed radiation detection devices and mobile radiation detection devices. Each device has the ability to self-configure and operate independently. However, these two types of devices can be configured remotely by TCP/IP through 3G/LTE modules to work together under the coordination of central control software. Fixed devices are sometimes used as a ‘tripwire’ to detect the presence of radiation, after which mobile devices are introduced as a secondary scanning method to confirm the presence and identify the source of the radiation. The architecture and function of the components in the IoRSS system will be detailed in the following sections.

### Detectors design

#### Detector selections

To take advantage of sensing techniques and sensors, the authors chose two different types of radiation detector when designing the system. First, because it is considered as a primary detection solution, the stationary device works all time to detect any abnormal radiation dose rate in the production area. Hence, its detector (primary detector) needs to be a long-life and durable one. Low-cost, small-profile, and simple circuitry are also additional requirements in practical implementations. Among the various types of radiation detector, Geiger-Müller (GM) tubes can meet almost all the mentioned criteria. Second, when there is an alarm in the stationary device, the secondary detector in the mobile device is used to confirm the appearance of the radiation source and identify the radioisotope. The most important requirement for the secondary detector is the ability to measure the energy of ionizing radiation with good resolution. Its size and mechanical strength should be suitable for integration into a handheld device. This leads to the use of a scintillator, in particular a CsI(Tl) (Thallium-activated cesium iodide) scintillator^45^. The CsI(Tl) crystal has not only good stopping power but also good plastic mechanical properties. Because this material can give a light output of 54 photons/keV and most of its emission is long-wavelength (>500 nm), the combination between a CsI(Tl) scintillator and a photodiode has become a good solution for the mobile device.

#### Detector circuits

**Figure 2:**
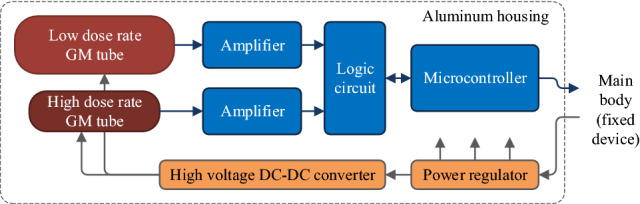
Structure of the primary detector module with the GM tubes.

In the primary detector module, a unique cylindrical aluminum box is used as the housing for two GM tubes, amplifiers, digital circuits, and power regulators, as shown in Fig. 2. The authors used two different types of GM tubes (71320 and 716, LND; or 70035 and 70018A, Vacutec) to ensure the good detection ability of the detector at low and high dose rates. The GM tubes are powered by a 550 V source created by a switching DC-DC converter. Because the signals from the GM tubes are weak analog pulses, amplifiers and logic circuits need to be used to amplify the changes and convert them to square waves. The microcontroller (Atmega128, Microchip Technology) controls the logic circuit, counts pulses, processes the data, and sends the result to the main body of the stationary device. Figure 2 shows the structure of the primary detector module with the GM tubes.

**Figure 3:**
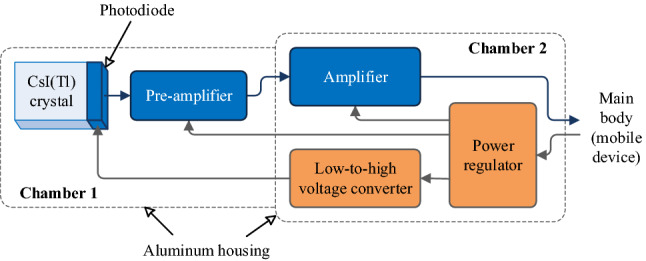
Structure of the secondary detector module of the CsI(Tl) scintillator.

**Figure 4:**
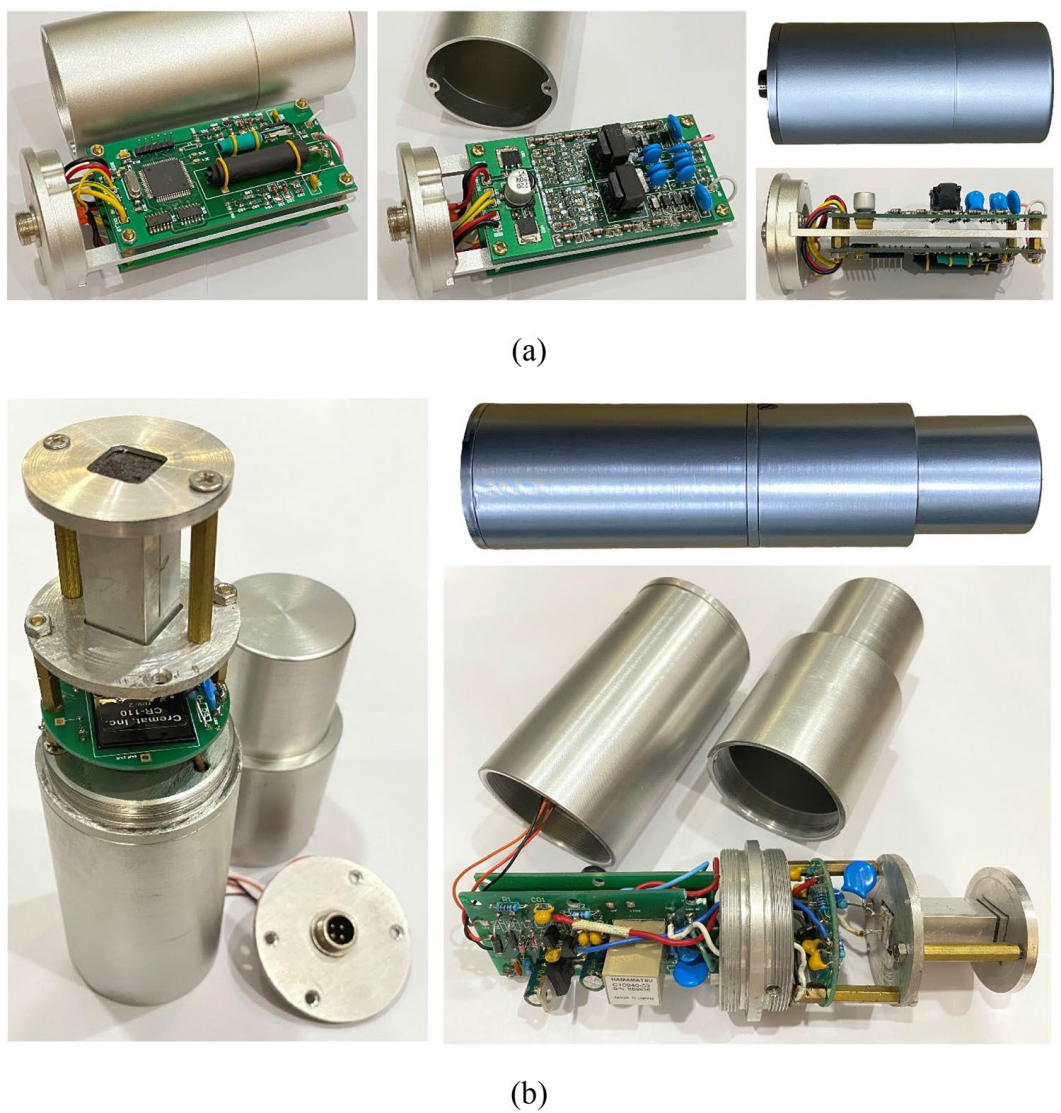
Detector prototypes: (**a**) detector module with GM tubes and (**b**) detector module with the CsI(Tl) scintillator.

The secondary detector module consists of a scintillator, a photodiode, an electronic circuit, and a 2-chamber aluminum housing, as shown in Fig. 3. The CsI(Tl) scintillator, size of 1 × 1 × 3 cm or 2 × 2 × 3 cm, and the avalanche photodiode^45^ (S8664-55 or S3204-08, Hamamatsu) are glued together using optical resin (OE-6662, Dow Corning). The photodiode is biased by a 380 V source created by a high voltage power supply module (C10940-53, Hamamatsu). Because the photodiode signal is ultrasensitive, a dedicated preamplifier is needed (CR110-R2, Cremat); and this amplifier must be completely separated from the remaining electronic circuit. Hence, in the aluminum housing, chamber 1 contains the scintillator, photodiode, pre-amplifier, while chamber 2 contains the further processing circuit and power modules. Figure 4a is a prototype of the GM tube detector module and Fig. 4b is a prototype of the CsI(Tl) scintillator detector module.

### Fixed radiation detection device (Fixed Device)

**Figure 5:**
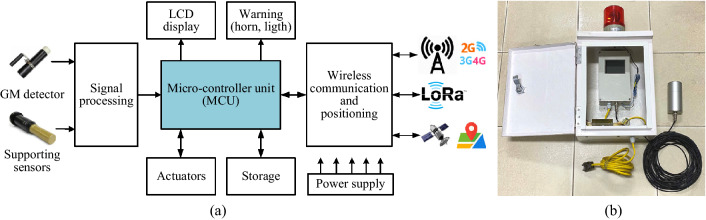
The fixed device: **a** architecture block diagrams and **b** an image of its prototype.

The architecture of the fixed radiation detection system consists of functional blocks as described in Fig. 5a.

- Wireless communication and positioning block includes a LORA transceiver and a GPS receiver. The block provides a wireless communication link from the fixed device to the gateway and localizes the position of the device.
- Signal processing block is a radioactivity detector system equipped with a GM tube system (Geiger - Muller tube), which can work in high-temperature environments such as around smelting furnaces at scrap metal recycling facilities. The radioactivity detector block is set to continuous operation mode with a detection range of gamma rays of about 5m and the detection threshold of 0.1 μSv/h. The block also includes supporting sensors consisting of a temperature sensor, accelerometer, and vibration sensor. They are designed to detect and transmit ambient temperature, vibration, and vehicle speed to the central processing unit. This data is used to adjust the working mode of the device.
- Warning and LCD blocks are designed to turn on the primary warning lights and horns and display data on the LCD screen when the radiation level exceeds a predefined threshold.
- Power supply block operates on an AC input voltage and generates DC output voltages power for function blocks in the device.
- Micro-controller unit (MCU) controls all operations of the fixed device, in which, the MCU receives data from the radiometric detector block and the sensor block, performs analysis, stores the data, and then displays the results of radiation levels on the display screen. The MCU turns on warnings with lights and whistles when detecting radiation levels that exceed a predetermined threshold. The MCU encapsulates data, including radiation level, vehicle speed, device location information into packets, and controls the wireless communication block to send the data packets to the gateway (using LoRa module) or to the monitoring center (using 3G/LTE module).

All components of the fixed radioactivity detector are integrated and enclosed in a protective enclosure, accompanied by mounting mechanical devices suitable for the operating conditions. Figure 5b is the image of the actual fixed radioactivity detector.

### Mobile radiation detection device (Mobile Device)

The mobile device is used separately to accurately detect the location of a radiation source or is used coupled with the fixed device to confirm the existence of the radiation source after a primary warning from the fixed device is activated. This device can be considered a secondary detection and warning device as it provides more specific information about the radiation source when detected and can be used to detect radiation in special cases such as hidden corners, shielded locations, open and scattered spaces outside the factory.

**Figure 6:**
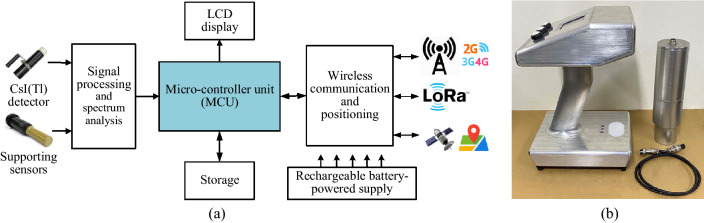
The mobile device: (**a**) architecture block diagrams and (**b**) an image of its prototype.

The architecture of the mobile radiation detection system consists of functional blocks, as described in Fig. 6a.

- Wireless communication and positioning block are designed similarly to that block in the fixed device. In addition, this block provides the ability to connect and exchange information directly between mobile and fixed devices in coordinated operation mode. The GPS receiver on the mobile device allows locating the radiation source. The location will be handled on the server and accurately displayed on the map that helps the monitoring to occur continuously and effectively.
- Signal processing and spectrum analysis block are equipped with a CsI(Tl) scintillation detector and a gamma and neutron spectrum analysis module allowing the measurement of radiation sources with energies from 0.03 MeV to 3.0 MeV.
- Micro-controller unit (MCU) controls all operations of the mobile device, in which, the MCU receives data from the signal processing and spectrum analysis block and the supporting sensor block performs analysis, stores the data, and then displays the radiation levels results on the display screen. The MCU encapsulates and transmits data to the gateway or shares the data with the fixed device through the wireless communication block. The encapsulated data include the instantaneous dose rate, the average dose rate, maximum dose rate, identified radiation sources, and the location of the sources.
- The rechargeable battery-powered block allows the device to operate continuously for up to 24 hours in the none warning scenario and 8 hours continuously in the warning scenario.

The mobile device is designed with compact size and can be used as a handheld device as shown in Fig. 6b. The highly flexible device is suitable for checking challenging positions, especially in hidden corners and tight crevices, to accurately determine the location of the radiation source.

### Gateway device

The gateway device acts as a centralized station to receive data from radiation detection devices and then perform data aggregation and data forwarding functions to the operation and control center (Server). The gateway also receives control data and configuration commands from the Server and then forwards these commands to the corresponding radiation detection devices. The gateway is integrated with multi-radio communication platforms, such as 3G/LTE and LoRa, to communicate with radiation detection devices. In addition, the gateway is also integrated with WiFi and Ethernet modules to ensure reliable communication with the server.

**Figure 7:**
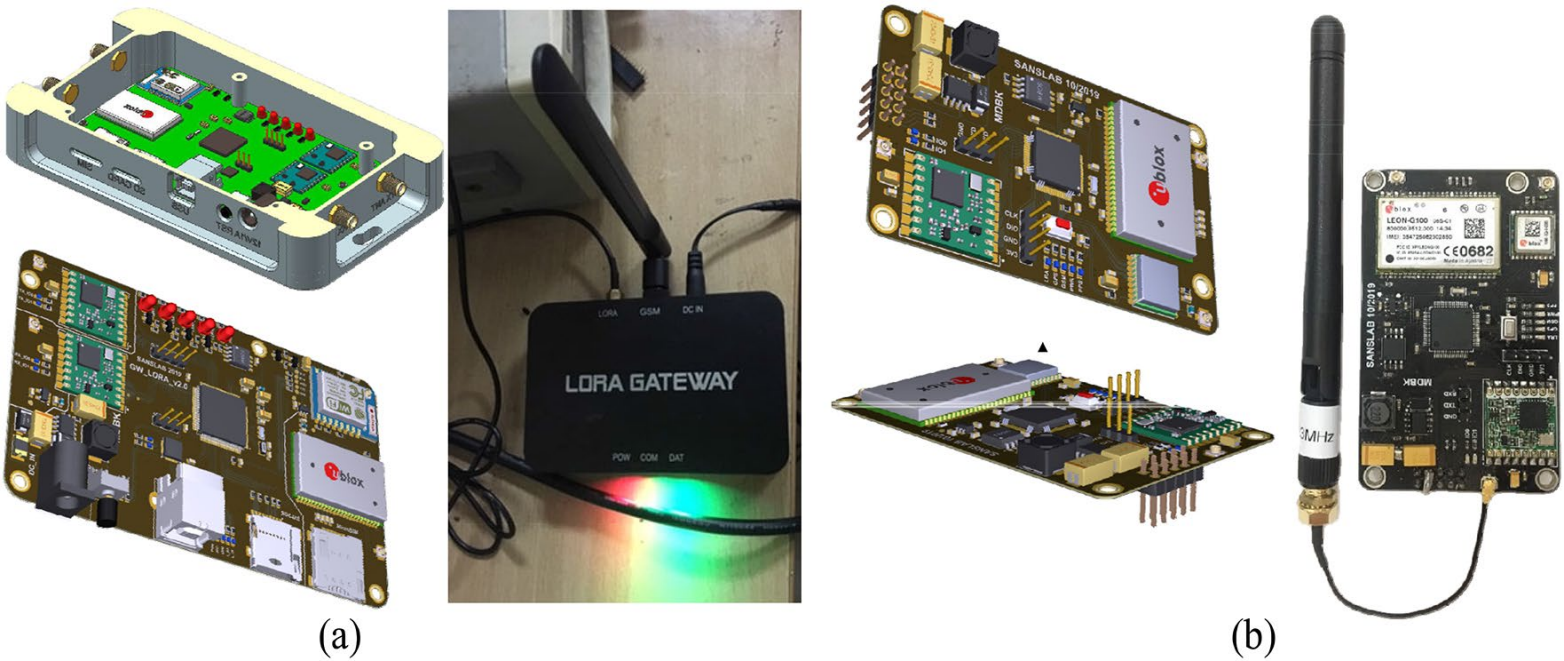
Layouts and prototype images of (**a**) the gateway and (**b**) LoRa, 3G/LTE, and GPS modules equipped for mobile and fixed devices.

Figure 7a is the layout design and the actual image of the gateway device and Fig. 7b is the layout design and the actual image of the integrated communication module LoRa, 3G/LTE, and GPS receiver. The integrated communication module is used on all gateway, the fixed radiation detection device, and the mobile radiation detection device. Within the limited scope of this paper, we do not show in detail the energy-efficient LoRa communication protocol specifically designed for this radiation detection system.

### Monitoring and control center

The monitoring and control center includes a system of server services, management tools, monitoring and warning software, handling processes, and radiation detection and warning procedures. Their components and functions are as follows.

- Network and cloud servers are responsible for communicating with radiation detection devices through the gateway or directly communicating with radiation detection devices through the 3G/LTE mobile communication network infrastructure. The network and cloud servers are also responsible for sending control commands from the users to devices.
- Database server is responsible for storing data received from radiation detection devices through a network server. The database server is also a place to organize and store the system and user databases according to the designed management hierarchy.
- Application server is the center of the monitoring and control subsystem. The application server provides system administration and management tools, data monitoring, data processing models, warning models, and radiation incident response. The application server creates an environment that allows users to interact with the system and provides services and data for user applications.
- Desktop app, the mobile app, and the web app provide real-time environment monitoring, control system, and configure device operation modes to review the history of detection parameters and search for device location on a digital interactive map. The user application software also provides interfaces that allow users to interact and operate the system, such as creating and sending control commands, creating commands to configure device operating modes, configuring processes radiation detection and warning services.

## IoRSS operation protocols

Based on the IAEA technical guidelines for dealing with the problem of controlling orphan sources and other radioactive materials in the metal recycling and production Industries^1,4,6^ and our actual survey results at metal recycling and production facilities in Vietnam, in this section, we propose processes of radiation detection, identification, and warning. These processes are designed, developed, and integrated into the IoRSS system mentioned in “System architecture and hardware design for an IoT-based radiation sensor system (IoRSS)” section.

### Objects management and configuration

**Figure 8:**
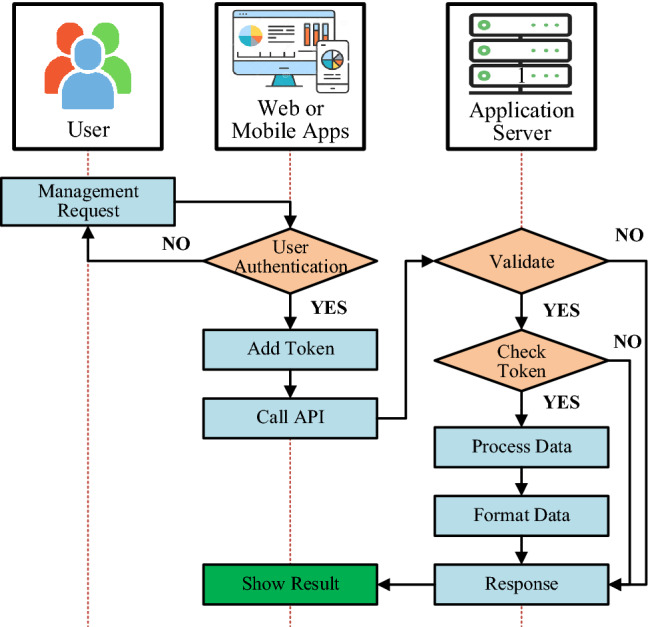
Flowchart of object management and configuration in the IoRSS.

Objects in a radiation incident response plan include human, material and specialized equipment. The specialized equipment in the proposed IoRSS system is the fixed and mobile radiation detection devices, gateway, and server service system. The objects involved in the radiation incident response plan can be managed and configured by functional software with the activity flowchart described in Fig. 8.

### Radiation detection and warning procedures

**Figure 9:**
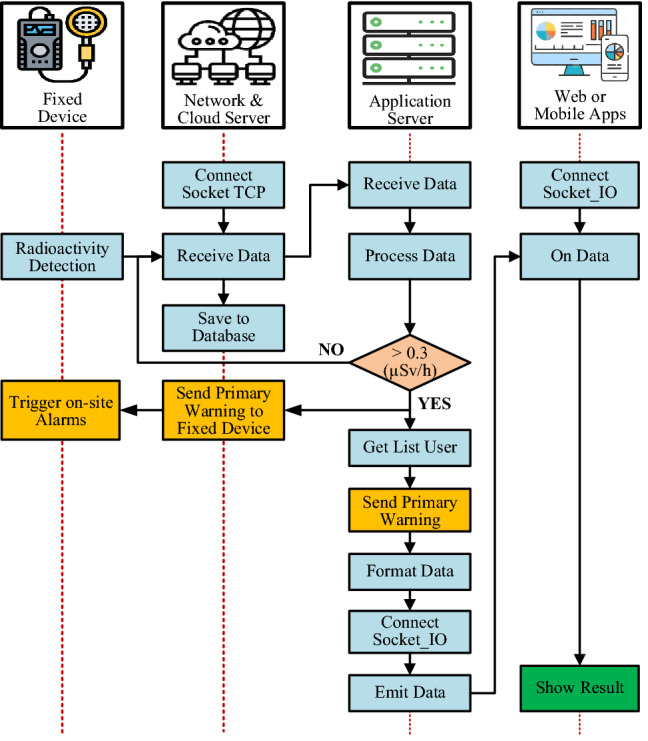
Flowchart of radiation detection and primary warning activation on the fixed device.

The algorithm to detect and monitoring radiation sources to trigger a primary warning level of the fixed radiation detection device is shown in the flowchart in Fig. 9. In this algorithm, the fixed device plays a role in continuously detecting and measuring radiation parameters and sending it to the server via a gateway using LoRa radio communication technology or transmitting directly to the network server via a 3G/LTE mobile communication network infrastructure. After receiving data, the network server stores the data on the database server. At the same time, data is also sent to the application server for processing, analysis, and providing real-time monitoring services for application software.

**Figure 10:**
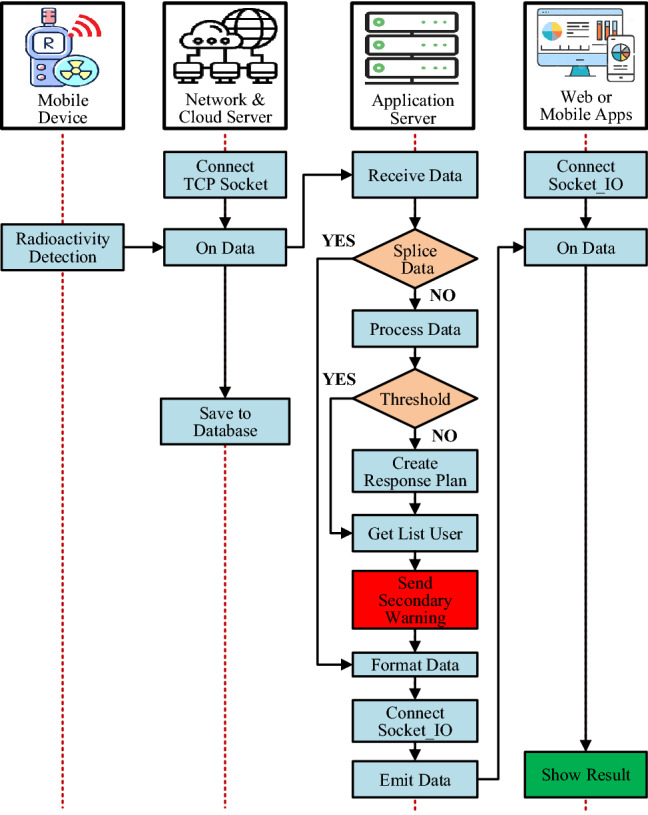
Flowchart of radiation confirmation and secondary warning activation on the mobile device.

The application server provides an online monitoring map with the following information: current value, the average value over a given period of time, and maximum value of measured radiation, device location and measurement time. The application server also performs data analysis algorithms on the received data. In case the measured value exceeds a predefined threshold, the application server will generate a primary warning level so that users can use the mobile device to confirm the existence of the radiation source, identify the type of radiation, the type of radioisotope (based on the spectral graph analysis algorithm), and the exact location of the radiation source. When the main warning level is initialized, the application server also sends a command to control the corresponding devices to turn on their local alarm with a loudspeaker, buzzer, or flashlight. Speed warnings can also be generated when a scrap metal transport vehicle is detected to move too quickly through the fixed radiation detection device.

When the primary warning is received from the system, users will use the mobile device to check and re-confirm. The measured parameters from mobile devices will continue to be sent to the network server and processed by the application server. Based on the confirmed radiation dose rate compared to the level of preconfigured thresholds, the system will activate an incident response procedure corresponding to the level of danger of the detected radiation source. The algorithm to confirm the radiation source and activate the radiation incident response process of the mobile device is shown in the flowchart in Fig. 10.

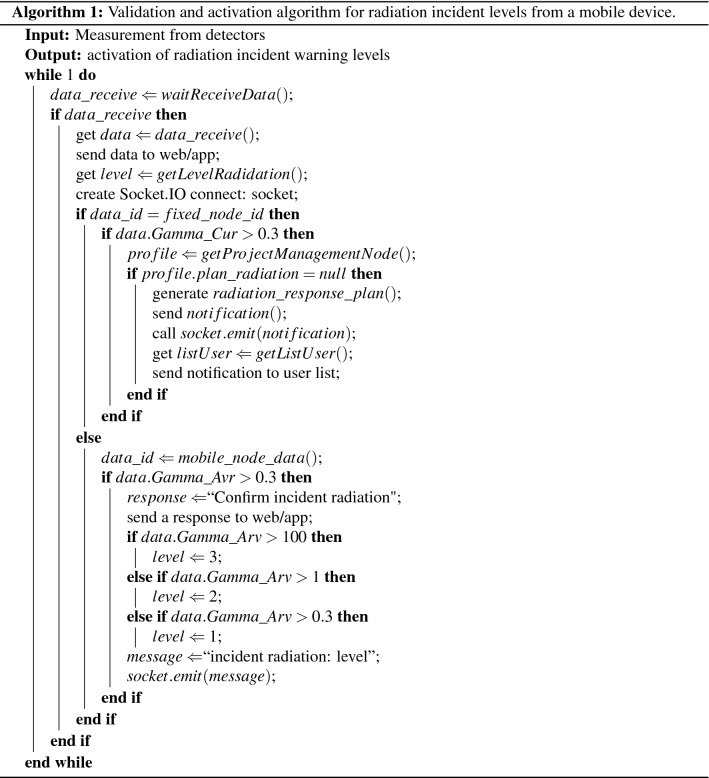



According to IAEA recommendations^4^, radiation incidents are divided into 3 levels: level 1 is dangerous when the measured values are in the range: 0.3–1 μSv/h; level 2 is a very dangerous level when the measured value is in the range of 1–100 μSv/h; level 3 is an extreme dangerous level when the measurement value is greater than 100 μSv/h. However, the threshold levels can also be adjusted correspondingly (by remote configuration command) to the environment background in each specific area. The algorithm to analyze and process data on the application server to trigger radiation incident warning levels is shown in Algorithm 1.

### Radiation incident handling procedure

**Figure 11:**
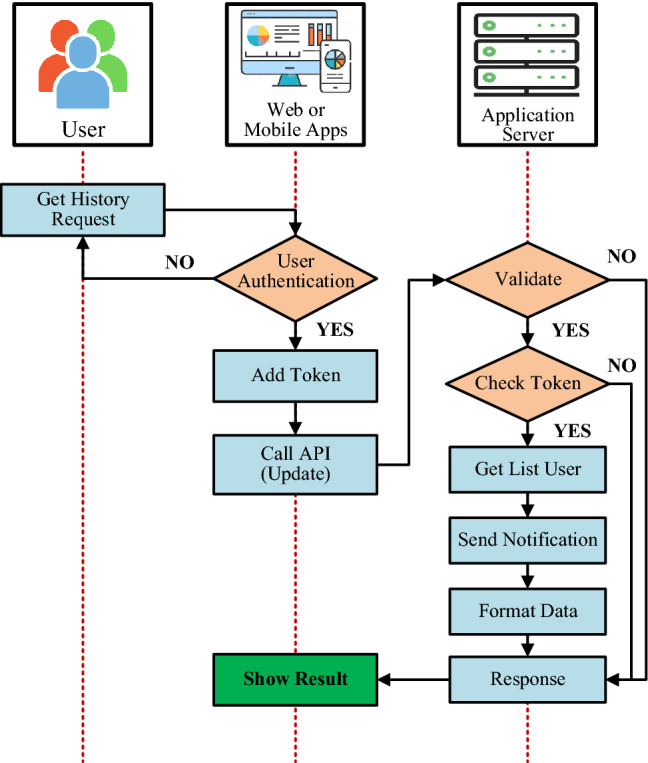
Flowchart of support information channels and radiation incident handling process.

During the radiation incident handling procedure, the person in charge is responsible for notifying and directing the relevant units to promptly handle the incident. The IoRSS system provides an interface that allows the administrator to create message contents and send them to the application server. The system will automatically send this information to relevant units and individuals by SMS and e-mail. Flowchart of creating and exchanging information to support radiation troubleshooting is shown in Fig. 11.

The IoRSS system also provides commands to control radiation detection devices and query radiation parameters remotely through the interface of application software. This mechanism allows the user to know the device’s status, configure the operating mode for the devices, and collect information about the operating environment of the device without having to approach the device on the assumption that the environment is radioactively contaminated.

### Radiation incident control and update procedures

**Figure 12:**
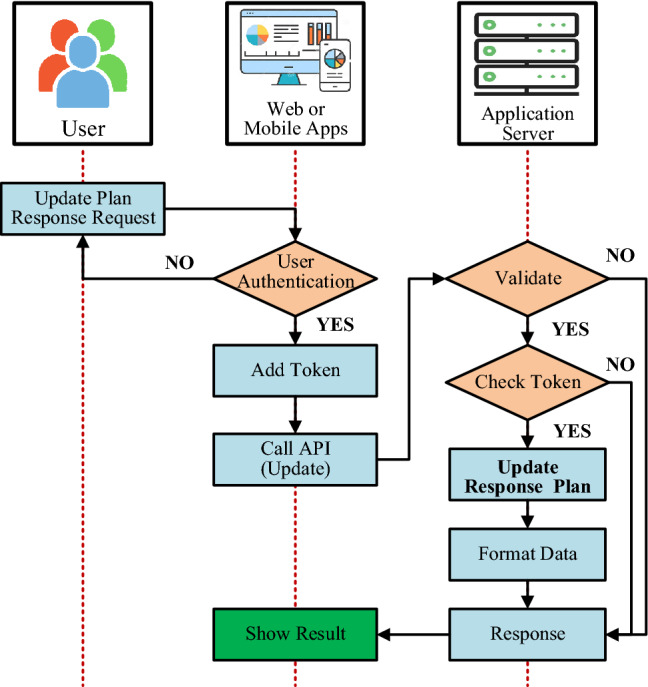
Flowchart of radiation incident control and update processes.

In the procedures of controlling and updating troubleshooting information, the administrator can also use the system’s interface to update information on the progress of control and troubleshooting, radiation source recovery, clean the environment and finish the problem according to the algorithm shown in Fig. 12.

### Web-based and mobile applications

In addition to the hardware design and communication protocol development, a real-time radiation detection and monitoring system includes software to monitor and provide the location and status of radiation detection devices periodically, continuously, or as requested. This software also provides operational functions, system administration, decentralization, statistics, reporting, and warnings about special situations. To achieve the designed functions and to facilitate future expansion and integration of the system, we designed the platform libraries to provide location-based services (LBS) that allow the implementation of update applications and rapid access to data in a synchronized process. The utility software is currently development: display positioning and surveillance in real-time software; system administration software for configuration of the operation and management mode (code, renew, active, inactive, etc.) of the devices; search and query data software; diagnostic utility, debugging, and system recovery; automatic alerting/warning according to each level via SMS, email, or call to the responsible people in case of warning events; utility surveillance logging and the providing instructions regarding how to handle special situations, such as the radiation dose rate exceeding the prescribed limit or the detection of radioactive material in scrap metal; and software showing the analytical and statistical data on the location, operating status of the device, radiation dose rate, the presence of neutrons, and battery power status in forms of charts and graphs.

**Figure 13:**
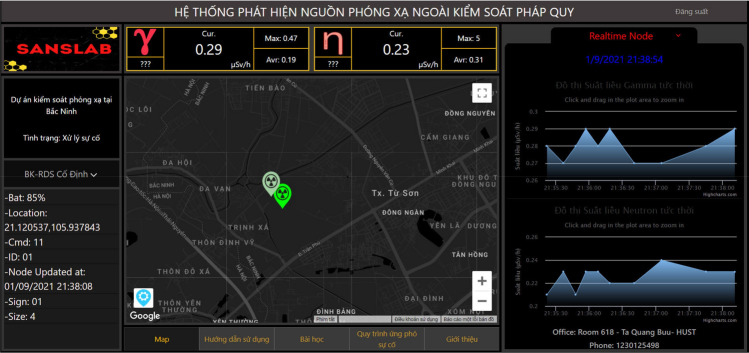
User application with a web-based interface, customized for Vietnamese users.

**Figure 14:**
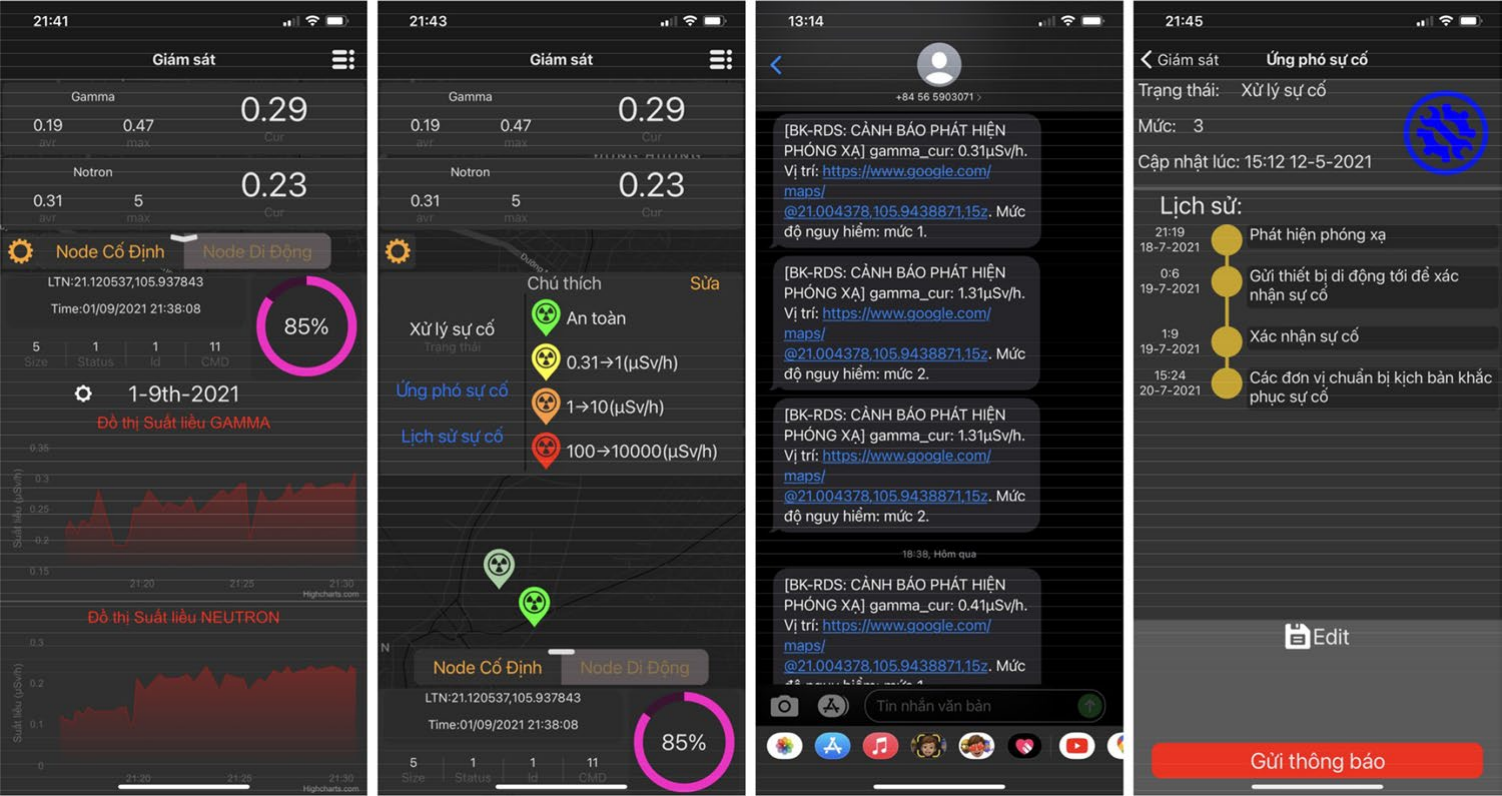
User application on a smartphone, customized for Vietnamese users.

Figures 13 and 14 are some images of web-based applications and smartphone applications, respectively, which show the functions of object management and configuration, radiation detection and warning, radiation incident handling, radiation incident control and update, and real-time radiation source surveillance.

## Test results and analysis

In this section, we present the results of extensive field tests in scrap metal recycling and production facilities to evaluate the performance of the proposal.

### Testing scenario and installation

Radioactive sources, if present in metal scrap, are usually shielded by metal materials, resulting in very low levels of radiation released into the environment. Therefore, to increase the probability of being detected, the location of the radiation detection system installation in this case is essential. Based on the actual survey results, we suggest some of the best installation locations, which are at the entrance of the incoming scrap truck, next to the weighing station, on the crane used to transport materials, next to the input / output slider, in the electromagnet used to lift and manipulate the scrap metal in the yard, or in front of the metal furnace door. Radiation detection devices installed at these locations would allow increased sensitivity in the detection of orphan sources while ensuring little impact on the production activities of the facility.

**Figure 15:**
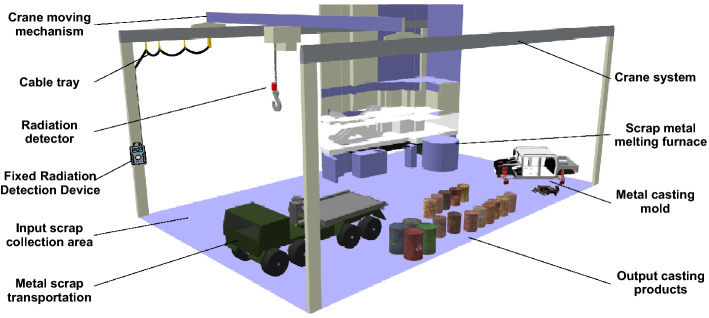
Diagram of the IoRSS deployment and installation in a scrap metal recycling facility.

**Figure 16:**
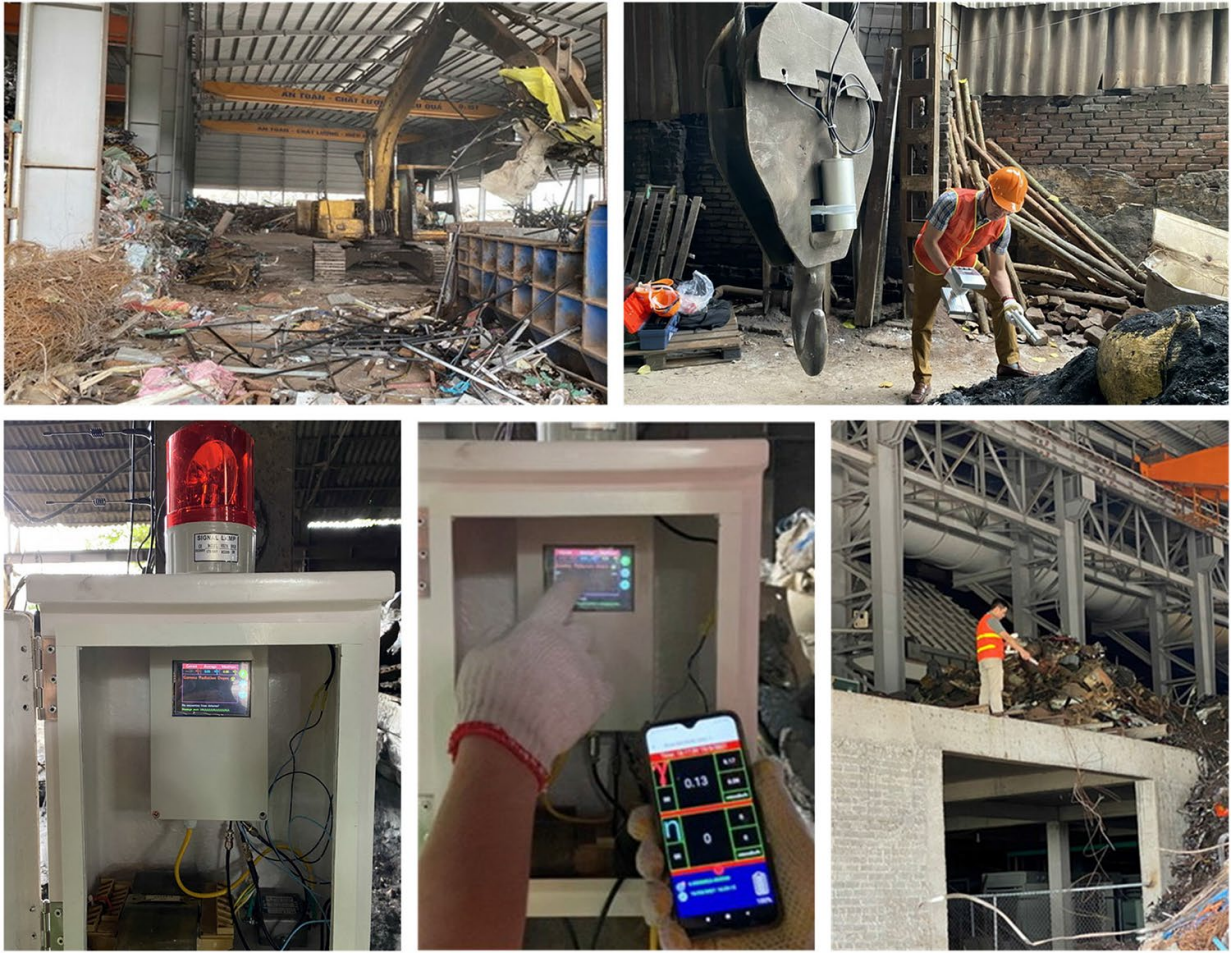
Testing the radioactive detection system in a scrap metal recycling facility.

Figure 15 illustrates a scenario for the installation a radioactive detection system in a scrap metal recycling facility. Consequently, a fixed radiation detection device is arranged on one side of the entrance to measure the radiation levels of vehicles that transport metal scrap when entering the recycling facility. The radiation detector can be installed on the crane hook, which is moved by the crane moving mechanism. The radiation detector is connected to the central processing unit of the fixed device via a cable arranged in the hanging chute. Crane system brings scrap metal from the transport vehicle to the input scrap collection area to prepare for putting into the furnace or transporting the output cast product to the output yard. According to this installation, the radioactive detector is always approached to the input scrap and the output product at the best distance to be able to detect the smallest radioactive source, if any, either in the scrap or in the product output (in the worst case, the ability to detect radioactivity at the input is missed). If radiation is detected, the fixed device will issue a local alert and send a primary warning to the server. Radiologists can use mobile devices to verify the primary warning and to accurately detect the location, level, and type of radiation source. In addition, cameras and accelerometers (not shown in the figure) can also be placed at suitable locations to record images and determine the speed of each vehicle when exiting and going to the recycling facility.

### Operation activities

After being assembled and dosed in the laboratory, the proposed system was tested several times in the field. The test configuration includes one fixed device and two mobile devices. The fixed device is installed according to the recommended scheme as shown in Fig. 15. Figure 16 includes images of a test system at a scrap metal casting facility. The results show that the radiation detection system works stably and does not affect the operation of the foundry. The main test activities and their results are shown in Table 1.

**Table 1:** List of test contents and achieved results of the IoRSS system.

Testing and assessment	Results and achieved parameters
Radioactive background measurement	Change around the value of 0.1 μSv/h
	Minimum dose rate: 0.05 μSv/h
	Maximum dose rate: 0.36 μSv/h
	Average dose rate: 0.12 μSv/h
Radiation detection and primary alarm generation functions of the fixed device	Display and update the measured value continuously on the screen; Trigger the main alarm when the measured value exceeds the pre-set threshold
LoRa communication connections	Send data and receive control commands with transmission delay less than 4.8 s
3G/LTE communication connection	Send messages directly to the network server; receive control command response and send ACK reply correctly
Operate the crane and observe the operation of the fixed device	The fixed device is still operating stably
Radioactive source identification and secondary warning generation functions of mobile devices	Radioactive sources (test samples) are identified by spectroscopy and the device generates a secondary alarm
GPS positioning function	Displays the correct current coordinates of devices on the digital map of applications
Energy consumption of mobile devices	Battery power drop after 1 hour of operation (measuring five times, alternating with standby state) is less than 2% of 3400 mAh × 2 rechargeable battery packs, the capacity loss is around 1.5%

### Performance of radiation detection

The fixed radiation detection device is standardized to a radiometric reference field (dose rate measurement) with standard gamma sources such as ^137^Cs, ^60^Co, ^133^Ba, ^241^Am, and ^228^Th. The mobile radiation detection device is a spectrum analyzer (counts recorded over time) and is also standardized with the above radioactive sources. The accuracy and reliability of radioactivity detection devices have been confirmed through actual tests. After the fixed device with dose rate monitoring system detects a change in the radiation field (increased dose rate) and provides a primary alarm, the mobile device is brought in to determine the type of radiation source and its activity source by analyzing the radiation spectrum recorded on the device display.

To evaluate the ability to detect radioactive sources of the proposed IoRSS system, we tested a prototype system consisting of a fixed radioactive detector and a mobile radioactive detector at a scrap metal recycling facility in Bac Ninh province, Vietnam.

**Figure 17:**
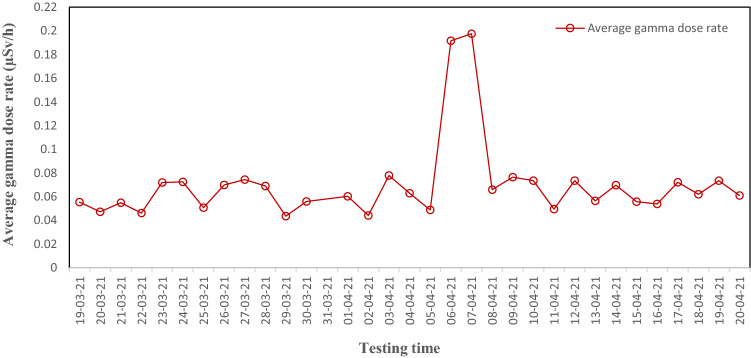
The measurement of the average gamma dose rate during the test period from March 19, 2021, to April 20, 2021.

Figure 17 is the result of the measurement of the average gamma dose rate during the test period from March 19, 2021, to April 20, 2021. It can be seen that the gamma value recorded from the IoRSS system varies with the average radiation background level of the test area (around 0.06 μSv/h). During the test period, the IoRSS system did not detect radioactive sources at the scrap metal recycling facility. The graph of Figure 19 shows that on April 6 and 7, 2021, there was a sudden increase in the average gamma radiation level (around 0.2 μSv/h). The reason is that during this time, the research team used a radioactive source with low activity (a radioactive source used in research) placed randomly in the metal scrap to check the ability to detect, warning, and identify the radioactive source of the IoRSS system. The test results showed that the IoRSS system accurately detected the location of the radioactive source, activated the corresponding radiation warning level, and recognized this radioactive source (^137^Cs).

#### Gamma source identification

The proposed system was initially designed for identifying three common types of industrial radiation sources that emit gamma rays: ^192^Ir, ^137^Cs, and ^60^Co. The gamma ray energies of these sources are 317 keV, 662 keV, 1173 keV, and 1332 keV, respectively^46^. In experiments, the authors used ^133^Ba (356 keV) instead of ^192^Ir because of the availability in the calibration laboratory. The gamma rays of these two sources have relatively close energy levels.

On the measured gamma-ray radiation spectrum, there will appear three peak regions corresponding to the above energies. Therefore, the algorithm to determine the names of radioactive sources is designed as follows.

- If there is a local peak in the range of 300-400 keV while the count at the 400 keV level is low, the radiation isotope is considered as ^133^Ba.
- If there is a local peak in the range of 600-720 keV while the count at the 800 keV level is low, the radiation isotope is considered as ^137^Cs.
- If there are two local peaks with energy levels that are higher than 1100 keV, there could be a ^60^Co source.

Determining the source name according to the energy region is more convenient, easier, and faster than having to adjust the machine, calculating the exact channel corresponding to the peak. Radiation spectrometers under operating conditions in the environment, near the heat source, often drift about 10%, so it is reasonable to take the peak region so that the peak of the spectrum, even if it drifts, is still in the selected region.

#### Neutron source identification

The source of neutrons used in industry is usually a Ra-Be or Am-Be source. Although they are neutron sources, these sources always produce an accompanying gamma radiation field. That is because the powder Ra, in addition to emitting alpha particles that form neutrons, also emits gamma radiation. The source ^241^Am emits a soft gamma of 59 keV, but the generated neutron interacts with the surrounding matter in response to capture radiation, so it still produces fluctuations in the dose rate of the gamma field. Neutron measuring systems in general have low recording efficiency (due to the small probability of neutron interaction with the detector) and the use of neutron loggers requires well-trained operators. However, there is a simple way to detect neutrons: Use a paraffin converter mixed with Bo powder. Neutrons slowed down by paraffin will be absorbed by B10 in Bo (which has an isotope ratio of 19.5%) and emit gamma of 478 keV. These gamma-recording scintillation detectors have this very high gamma-recording efficiency, close to 100% with a crystal thickness of 3 cm. This is an efficient way to detect neutrons. Specific spectral regions to identify the sources of ^192^Ir, ^137^Cs, and ^60^Co are independent of the 478 keV region, so the 478 keV region (taken from about 420 to 550) is used to confirm the presence of neutrons. Because the emitted gamma 478 keV is a large peak, the evaluation of neutrons is based on the following algorithm:

- No peaks of ^192^Ir or ^133^Ba, ^137^Cs, and ^60^Co (as the descriptor algorithm of the gamma source detection function).
- Counting rate increases in the 420 keV to 550 keV region.

Radioactive sources that need to be identified are those used in industry, medicine, and irradiation for food preservation and sterilization. These are sources of impact on human health if they are lost to the environment and they are objects that need to be detected and identified if they are mixed with scrap metal. Standard radiometer sources in the laboratory are not included in the detection of this system.

**Figure 18:**
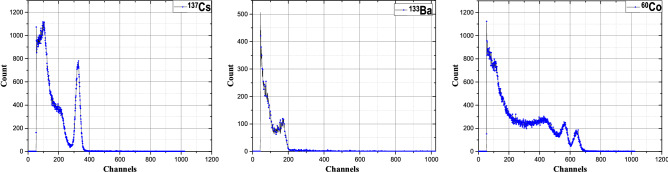
Spectral graph of radioactive sources detected by mobile devices (each channel corresponds to approximately 2.06 keV).

The performance and specifications of fixed and mobile radiation devices are listed in Tables 2 and 3, respectively. Figure 18 shows the energy spectra for some radioactive sources measured by mobile devices. The peaks in the graphs of ^137^Cs, ^133^Ba, and ^60^Co are around 670 keV, 345 keV, 1160 keV and 1345 keV, respectively. Comparing to the data in^46^, the corresponding errors are about 1.2%, 3.1%, 1.1% and 1.0%. The small shifts of the peaks have no effect when identifying the three radiation isotopes. However, when identifying sources with close energy levels, these errors may cause confusion. In this case, the possible solutions could be extending the capture time, applying a filter, or customizing the identification algorithm.

**Table 2:** Specifications of the fixed radiation device.

Parameter	Value
Energy range (Gamma)	0.05 to 3 MeV
Detection threshold	0.1 μSv/h
Detection response time	1 second
Probability of correct detection (tested with ^137^Cs, ^133^Ba, and ^60^Co)	99%
LoRa/3G/LTE wireless communications	Integrated
AC power supply	90 V to 240 V
Operation temperature	− 10 °C to + 50 °C

**Table 3:** Specifications of the mobile radiation device.

Parameter	Value
Energy Range (Gamma)	0.05 to 3 MeV
Neutron detection ability	Yes (Spectral Graph Analysis)
Dose rate range	0.1 to 123.2 μSv/h
Accuracy	30%
Identification of isotopes	^137^Cs, ^133^Ba, and ^60^Co
LoRa/3G/LTE wireless communications	Integrated
Battery lifetime (rechargeable)	60 h
Operation temperature	− 10 °C to + 50 °C

### Performance of LoRa communication

To evaluate the reliability of LoRa wireless communications and to ensure reliable data exchange and meet the real-time requirements of the IoRSS, we set up the following configurations and scenarios. One gateway is in a fixed place, and 1 to 15 mobile devices (with LoRa modules) are deployed around it as LoRa nodes. The distance from the gateway to the nodes ranges from 500 m to 3500 m in the urban environment. The spread factor, bandwidth, coding rate, transmit power, and size of the payload of the nodes are 10, 125 kHz, 4/8, and 17 dBm, respectively. We evaluate the packet loss rate over the number of nodes (network density), the distance of transmission, duty cycle (packet sending cycle), and packet length.

#### Packet loss over transmission range

**Figure 19:**
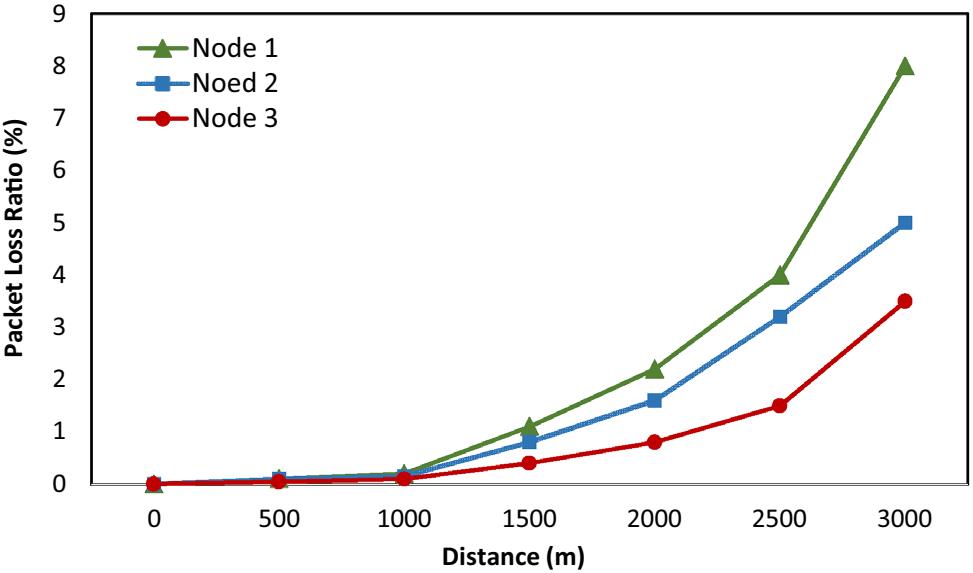
Average packet loss ratio as a function of transmission range.

In the first test, we sent 200 messages of 100 bytes in length every 30 s. As shown in Fig. 19, all nodes have the same shape of the graph, showing the relationship between transmission distance and packet loss rate. This means that the test nodes have good communication reliability if the distance is less than 2.5 km and the packet loss ratio starts increasing dramatically if the distance is more than 2.5 km. In theory, the transmission distance of LoRa ranges from 2 to 3 km in urban environments, and the results of this test have proven that our proposed system, in practice, still ensures the transmission distance, consistent with the theory. Although there is a high packet loss ratio when the distance is more than 3 km, we can reduce this ratio by using a higher gain antenna or by placing the gateway at a higher position to ensure line-of-sight propagation.

#### Packet loss over duty cycle

**Figure 20:**
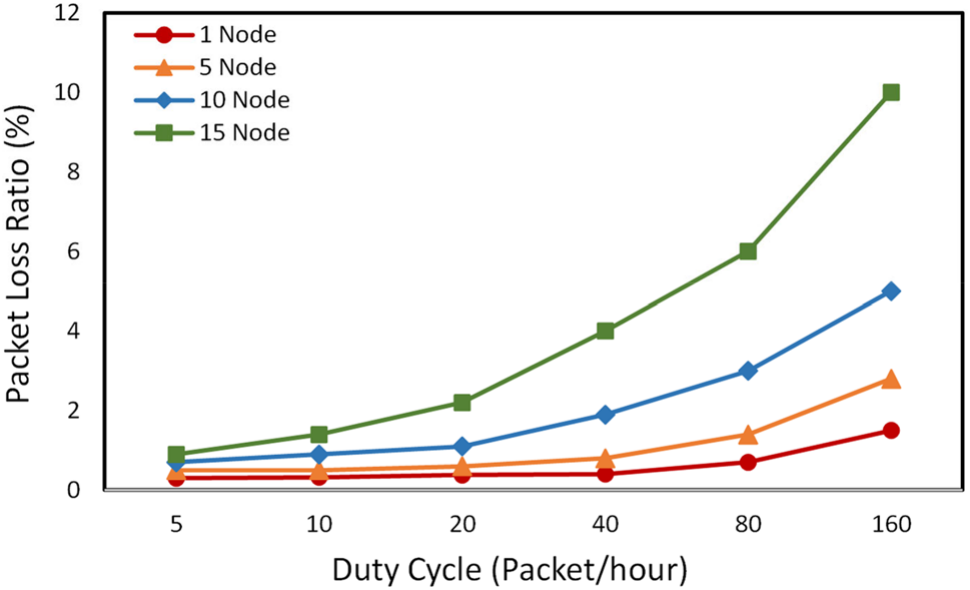
Average packet loss ratio as a function of duty cycles.

Figure 20 shows the relationship between packet loss rate, duty cycle, and the number of active nodes. In this test scenario, the size of each fixed-length packet is 100 bytes. As the number of nodes increases, the packet loss rate also increases. This is obvious because as the number of active nodes increases, the number of packets sent into the system increases, leading to an increase in the probability of packet collisions and an increase in the packet loss rate. However, when the frequency of sending packets is low, even though the number of nodes increases, the system still operates stably and reliably with a packet loss rate of less than 2%.

When the packet sending cycle is up to 160 packets/hour (i.e, one packet is sent every 22.5s on average), the packet loss rate is quite large, up to 10% and 6%, respectively to 15 and 10 active nodes. However, the LoRa communication protocol in this system is designed to operate with the acknowledgment (ACK) mechanism, so at a high duty cycle, it still ensures that the packets reach the gateway/server correctly. It should be emphasized that this is a test scenario with a transmission distance of up to 2.5 km. In fact, the operating range of the devices is normally a few hundred meters in the area of popular scrap metal recycling facilities. At this distance, the packet loss rate is almost zero, as shown in Fig. 19. If radiation detection devices need to operate outside of the coverage of the gateway, the 3G/LTE communication module on these devices will be activated to ensure a reliable connection between the devices and the server.

#### Packet loss over package length

The next test scenario evaluates the effect of packet length on system performance. As the packet length increases, the time to perform modulation at the node and demodulation at the gateway also increases. Additionally, increasing the size of the packet leads to increasing the time-on-air of the packet. Therefore, during packet processing, if another packet is also sent, the new incoming packet will be considered interference and will be discarded.

**Figure 21:**
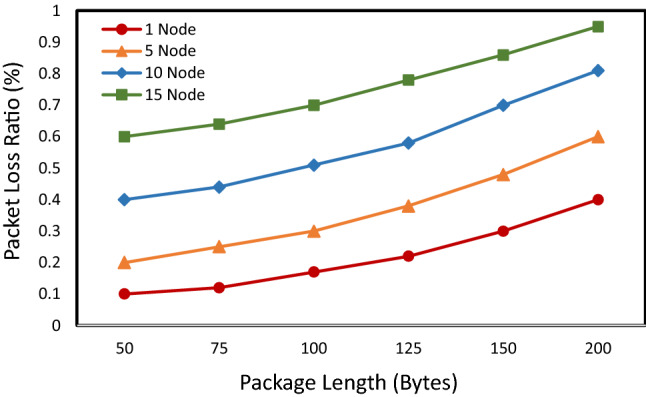
Average packet loss ratio as a function of package length.

Figure 21 represents the relationship between packet loss rate, packet length, and the number of active nodes. In this test scenario, each node sends five packets every minute. The results show that as the message length increases, the overall packet loss rate also increases. However, unlike the effect of the duty cycle, the package length has little impact on the performance of the system. When the message length is up to 200 Bytes and 15 nodes are active, the packet loss rate is only up to 0.9%. This result shows that the gateway achieves high performance under high load.

#### Latency and processing delay

**Figure 22:**
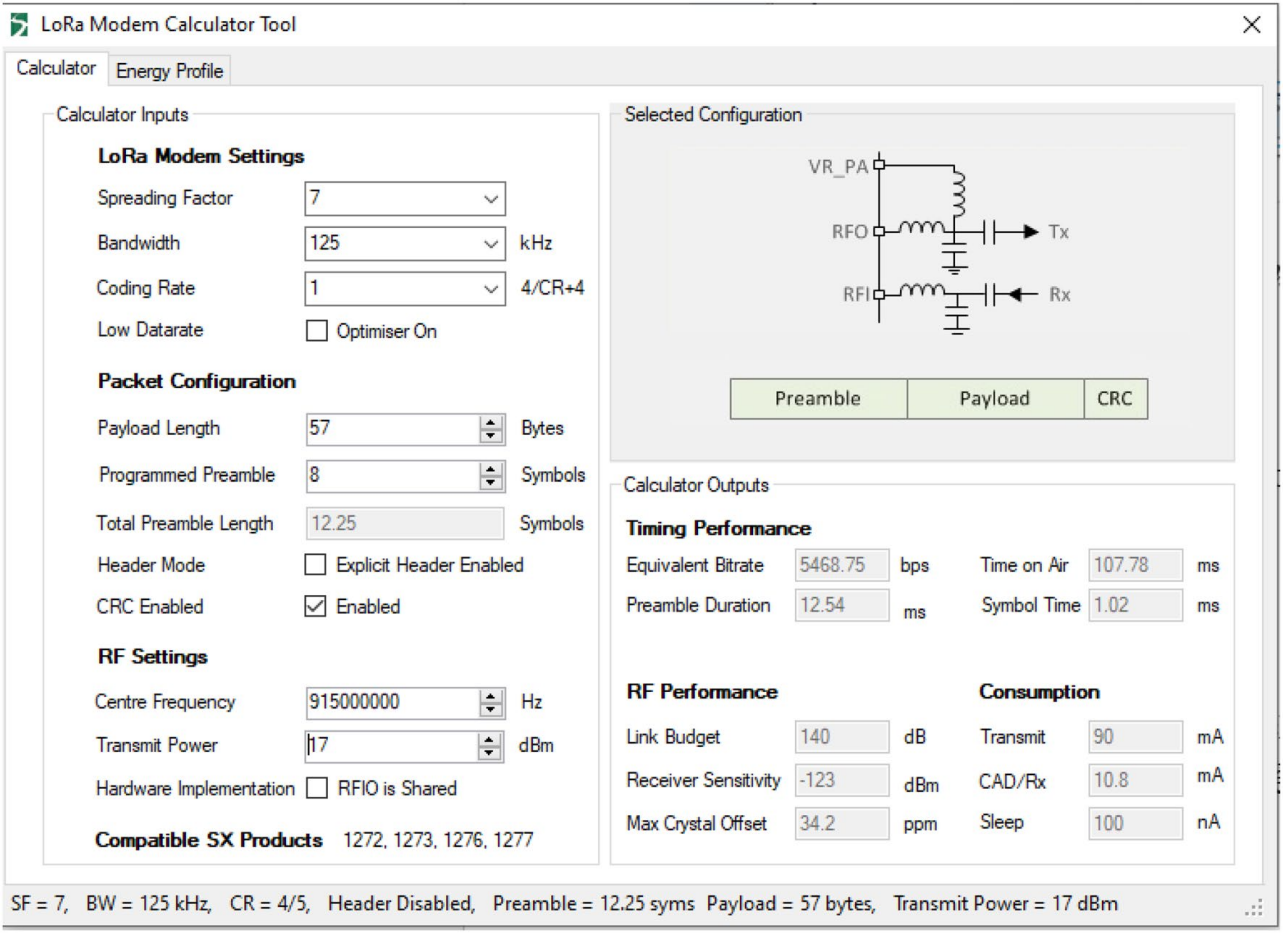
Calculation of ToA in the LoRa Modem Calculator Tool^48^.

In this subsection, we evaluate the latency and processing delay of radio communication between radiation detection devices and the gateway based on the time-on-air (ToA) parameter. Because the communication module of the radiation detection devices and the gateway are integrated with the LoRa SX1276 module, we use the LoRa Modem Calculator Tool^47^ to calculate, select, and configure the LoRa modem parameters, packet configuration, and radio frequency relevant to the proposed IoRSS system. The LoRa Modem Calculator Tool^47^ is an open-source software, freely provided by Semtech. This is a common tool used by many researchers for LoRa hardware development. Figure 22 is a screenshot of the LoRa Modem Calculator Tool interface that is installed on the HUST’ computer, with specific parameters selected in accordance with the proposed IoRSS system. For example, as shown in Fig. 22, the payload length = 57 bytes and the programmed preamble = 8 bytes correspond to the designed message structure exchanged between the radiation detectors and the IoRSS gateway concentrator in the IoRSS system (the designed message structure is not presented in this article due to space limitation), the selected frequency is 915 MHz, which is an unregistered frequency band in Vietnam, the transmit power is set at 17 dB, allowing reliable communication between wireless devices within a few hundred meters consistent with the size of typical metal recycling facilities in Vietnam. The parameters used to calculate latency and processing delay are referenced in the LoRa SX127x datasheet^48^. In the next test, we will measure and evaluate two parameters: the round trip time (RTT) and the processing time on the gateway. The RTT is the duration, measured in milliseconds, calculated from the time a node sends a message to the time it receives a response from a server. The formulation for calculating *RTT* is as shown in Equ. (1), in which $T_p$ is the propagation time, which is equal to the time-on-air (*ToA*) in this test, and $T_s$ is the processing time on the gateway.

1 $$\begin{aligned} RTT = 2 \times T_p +T_s = 2 \times ToA + T_s \end{aligned}$$

To measure the processing time $T_s$, we use the same configuration for the gateway and nodes as in the distance and reliability test scenario above. In this test, each node randomly transfers 100 bytes to the gateway. The distance between nodes and gateway is fixed at 1 *km*. The number of nodes ranges from 5 to 50. We capture the time when a node starts sending a message and the time when the node receives an ACK message from the gateway to calculate RTTs. We also capture the time that the gateway received messages from the nodes and the time that the gateway sent ACK messages to calculate the processing time on the gateway. The *ToA* parameters are calculated as the formulation shown in the SX127x datasheet^48^. In this test, we use the LoRa Modem Calculator Tool^47^ to calculate the exact value of the ToA parameter corresponding to the configuration in this test scenario. As shown in Fig. 22, *ToA* in this test scenario is 985.09 *ms*.

**Figure 23:**
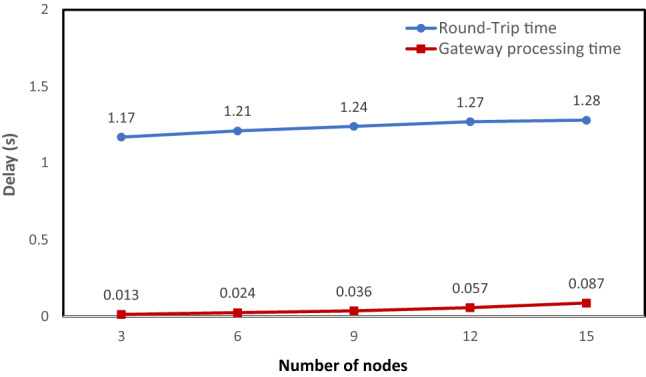
Latency and processing delay as a function of the number of nodes.

The results analyzed in Fig. 23 show that as the number of nodes connecting and sending data to the gateway increases, the processing time on the gateway increases, and therefore the RTT increases. This is obvious because as the number of nodes increases, the amount of data sent to the gateway increases, leading to an increase in processing time and resources on the gateway. However, in the proposed system, the processing time on the gateway ranges from 20 ms for 10 nodes to 250 ms for 50 nodes. This result is acceptable and absolutely satisfies the latency requirement (the latency is less than 300 ms in a common LoRa system with the same configuration).

#### Power consumption

**Figure 24:**
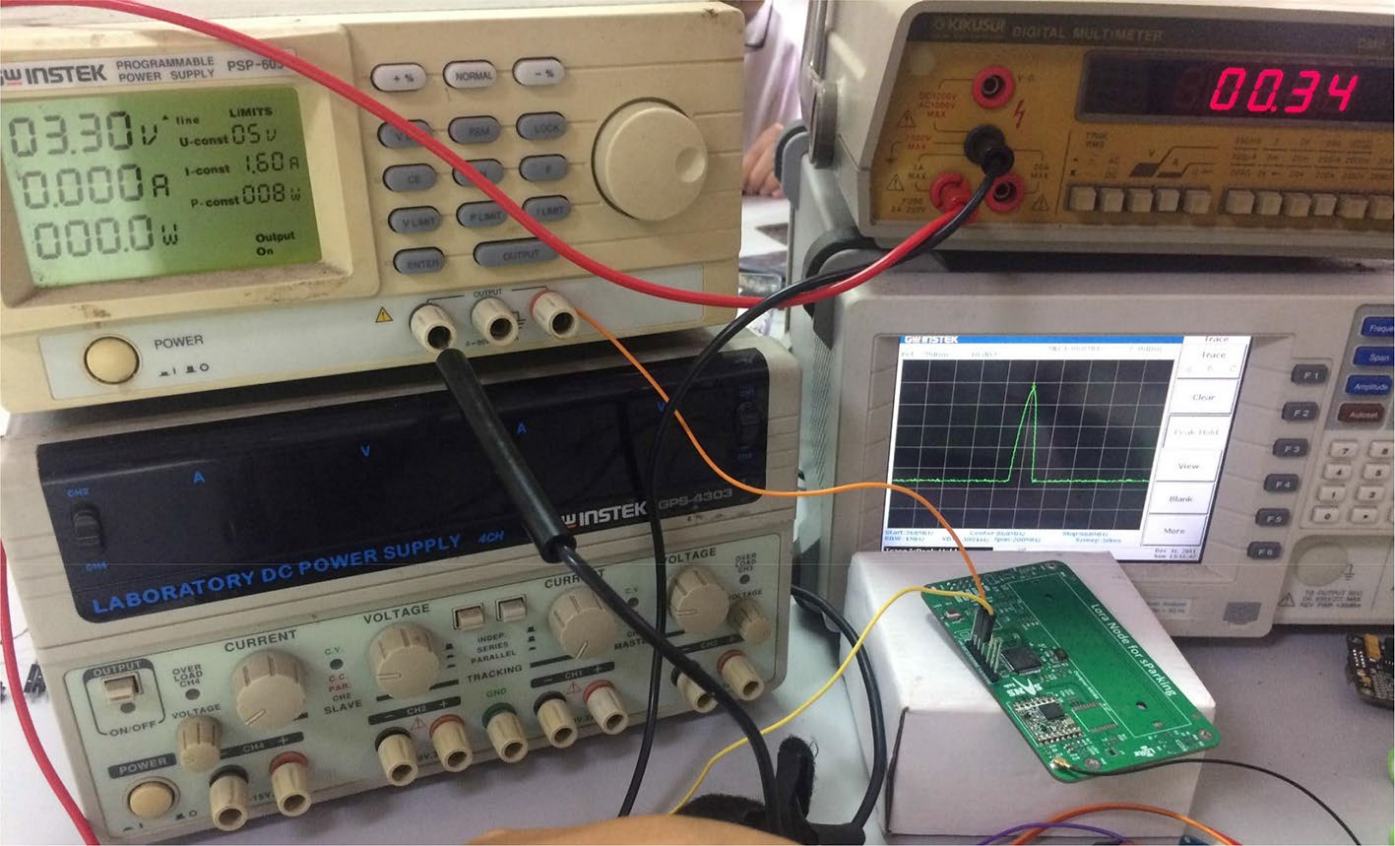
Lora node performance test and power consumption measurement.

The power consumption of a node depends on two main components: processor operation and LoRa module transceiver operation. In the proposed system, the node uses an STM32L072RBT6^49^ as the processor unit (MCU), and the LoRa module uses the RFM95^50^ chip for the radio transceiver. The results of theoretical calculations (based on the datasheet of STM32L072RBT6 and RFM95) and the results of practice tests show that in a normal operating mode (16 MHz), the node (only the MCU works) consumes approximately 1.8 (μA). In sleep mode, the current consumption is 0.35 (μA). For the Lora module, at short transmission distances, the current consumed when transmitting is 23 mA, and the current consumed when receiving is 11 mA. Figure 24 is an image of the LoRa node performance test and the power-consumption measurement. The experimental results are compared with the theoretical calculation results published in the design documents of the LoRa transceiver module (LoRa SX1278 module using the RFM95 chip) and the microprocessor unit (STM32L072RBT6) to demonstrate the performance of the hardware designed and prototyped in this work. Table 4 summarizes the measurement results.

**Table 4:** Compare the energy consumption of LoRa module according to theoretical calculations and practice tests.

Description	Theoretical calculations	Practice tests
Standby mode with RTC (MCU only)	0.29 (μA)	0.35 (μA)
Listening mode (crystal oscillator enabled)	1.6 (μA)	1.8 (μA)
Transmission mode with impedance matching (Tx)	20–120 (mA)	34 (mA)
Receive mode with LnaBoost On/Off, higher/lower bands (Rx)	10.8–12.1 (mA)	11 (mA)

### Results discussion

In this section, the authors discuss some important issues regarding the performance and applicability of the proposed system. The discussion points include the evaluation of the current results, limitations, and possible applications; the cost of the system and the trade-off cost-efficiency; the need for more intensive tests under real working conditions; and future works.

In this study, the authors have proposed a complete internet of radiation sensor system. The work is an applicable experimental design for a technical problem that is really meaningful to society. Among the key results of this study, the radiation detection hardware and the IoT system are the most important. First, regarding the radiation measurement and gamma ray energy resolution, compared with the similar study^12^ that used a larger detector, this study achieves a relatively good result. The energy spectra of the reference study are a little better than that of the proposed study; this could be due to the bigger size of the scintillation crystal. However, the detectors in both studies are excellent for identifying the common industrial radioactive isotopes. Second, the IoT network and protocol of the proposed system are constructed based on robust designs. This could be one of the first low-cost and regional IoT systems in the field of radiation detection for scrap metal recycling. Therefore, the authors measured the communication parameters rather than comparing the proposed IoT system with a reference design. Although this could be a limitation that should be addressed in the next study, the proposed IoT system is well suited to the requirements for connecting the devices in the proposed system.

Because the whole system is fully modularized, the proposed IoRSS can be easily customized not only detectors and other hardware but also to the IoT network and protocol. This makes the system more flexible and feasible. The proposed design could be a good choice for developing countries to follow the recommendations of the International Atomic Energy Agency. The system is also a solution for recycling factories to upgrade the ranking of their products by equipping radiation safety control devices. This means that the proposed system benefits not only the metal recycling plants, but also the entire society.

The cost of the system may not be a big problem when it is widely equipped for many scrap metal recycling facilities. The main reasons are: (i) the cost of the proposed system is not very high and will be strongly reduced in mass production; (ii) both the investment costs and revenue of metal recycling factories are often large; and (iii) there are regulations on the responsibility for detecting radioactive sources for scrap metal recycling^4^. Regarding (i), the cost of the entire system depends mainly on the radiation detectors, the hardware units, and the IoT system. In the proposed configuration, the use of two well-known kinds of radiation detectors makes the cost less effective than single detector systems. However, the system architecture and design of the system allow several factories to share the same mobile device and the same IoT system. This utilization could be a good solution for developing countries, where many small- and medium-scale metal recycling facilities are distributed in the same region. When considering (ii), the authors compare the cost of the proposed system (units of ten-thousand USD) and the investment costs (unit of millions USD) and the revenue (unit of millions USD) of the medium-scale scrap metal recycling facilities. In small-scale factories, the proposed system could be luxury equipment; however, there is a fact that the output of small-scale facilities is commonly the input of the larger factories. Thus, the installation of large- and medium-scale metal recycling factories still has meaning for society. Furthermore, when all countries force all metal recycling plants to be equipped with radiation detections, as mentioned in (iii), the small increase in a production cost will be a worldwide issue; hence, it is not a problem for any specific factory.

In addition to the financial issue, there is a trade-off between the investment cost, the radiation detection capability, and the response speed. The first point here would be the density of the radiation detection devices in the IoRSS system compared to the effectiveness of the detection and the costs associated with that density. To increase the probability of radiation detection in a large area requires a large number and density of devices to be installed. The detection efficiency also depends on the type and size of the radiation detectors; the more advanced and larger detectors exhibit better performance^41^. However, in this study, no economic analysis has been done to understand the relationship between the money spent on devices and the probability of detection.

The next point here would be the relation between the nuisance alarm rate and the response speed of the system. The stationary device is configured to update the dose rate every 1 second. However, in order to reduce the nuisance alarm at low dose rates, the software algorithm requires up to 5 seconds for confirmation. Even when the alarm is unstable, more time is needed to put the mobile device into operation. The software algorithm can be easily set up for responding faster; however, a high background dose rate may trigger a false alarm and disturb the operation of the factory. Hence, in terms of response speed, the proposed system is not real-time as the generic meaning. However, compared to the lead time of metal production, this speed is sufficient to detect and isolate the risk of recycling radioactive materials.

To fully validate the proposed system, more intensive tests under real working conditions. Performing more experiments in scrap metal recycling factories could make the proposed system more complete and more practical. Furthermore, testing the system under identical conditions helps the authors isolate association effects. Although this could be a current limitation of the study, it is not easy to perform many experiments in scrap metal recycling facilities. The reason is that in developing countries, scrap metal recycling commonly pollutes the environment; in particular, polluting the air when melting the scrap metal. Therefore, facility owners are wary of all measurement-related research in their factories, regardless of explanations. Therefore, researchers had few opportunities to perform the experiments under the desired working conditions within the factories. To overcome this challenge, support and legal permissions from governments are helpful.

The applicability of the IoRSS would be expanded if the measurement hardware is significantly improved in size and cost; the IoT communication system is validated with larger scales under harsh environmental conditions; the whole system is intensively tested in various types and scales of metal recycling facilities. Furthermore, conducting more studies to address the trade-off between nuisance alarm and sensitivity would make IoRSS more effective.

## Conclusions and future work

In this paper, we have designed and developed the Internet of Radiation Sensor System (IoRSS) to detect radioactive sources out of regulatory control in scrap iron and steel recycling and collection facilities. The IoRSS consists of a network of wirelessly connected stationary and mobile radiation detection devices using the reliable LoRa radio communication protocol. The radiation detection device, based on the combination of GM tubes and CsI(Tl) scintillators, was extensively tested under multiple conditions, including operation at high temperature, high acceleration, and in a high magnetic field in real scrap metal recycling and production facilities. The test results show that with the proposal of algorithms, approaches, software tools, specifications for data integration, and automation of the information processing to optimize the limited resources, the IoRSS provide a more robust detection capability and improve the use of data analytic in the assessment of radiation incident detection and response. Furthermore, building a radiation detection system based on an IoT system saves a large amount of energy, increases data transfer rates, and reduces latency.

The main technical characteristics of the proposed IoRSS system can be summarized as follows:

- Communication: Using low power LoRa radio communication technology to communicate between radiation detection devices and the gateway station. Using mobile communication network infrastructure to communicate between the gateway and control center (cloud/server).
- Location monitoring: Provide and display an accurate location of the device with a GPS positioning accuracy of around 5 m in good weather and environmental conditions.
- Radiation source detection and identification: Provides information on the number of radiation pulses and dose rate at the measurement site, and triggers an alert when radiation exceeds a given threshold. Able to identify radioactive sources on the basis of a spectral graph analysis function.
- Mechanical structure: The device has a mechanical structure suitable for installation in small and medium-sized scrap metal and steel recycling facilities. The mounting method ensures safety for the device and does not affect the normal operation of the facilities. The physical size of the mobile device is suitable for hand-held use.
- Control and configuration: The device has the feature of remote configuration setting/query via SMS (configuration setting / query by mobile phone message) or TCP/IP (using the web interface).
- Radiation incident response and warning procedures: The IoRSS system provides warning levels and activates radiation detection and incident response procedures corresponding to the danger level of the radioactive source being detected.
- Battery powered: Mobile devices powered by rechargeable batteries. The average operating time of each full battery charge is 3 days.
- Monitoring software: The software works stably with the functions of decentralizing access, statistical reports, online and real-time monitoring, remote control and configuration, and high-security features.

In the future, the IoRSS system will be expanded to be compatible with several other nuclear detection technologies and connected to form an intelligent monitoring and surveillance system. Various types of sensors and detectors will also be integrated into the system to enable the detection of other types of hazardous materials such as explosives, biological agents, and weapons. Incorporating stationary sensors and detectors of varying types mounted on a range of portable platforms such as Unmanned Aerial Vehicles and hand-held devices could dramatically improve the IoRSS effectiveness and can be readily implemented into our existing operation process and devices. The next foreseen study will be focus on mitigating some of the challenges related to IoT-based networks such as heterogeneity of devices, processing of heterogeneous unstructured big data generation by sensors and data security. We will also develop advanced algorithms and data processing models using artificial intelligence to provide smart services and applications.
